# Comparison between clinician and machine learning prediction in a randomized controlled trial for nonsuicidal self-injury

**DOI:** 10.1186/s12888-024-06391-x

**Published:** 2024-12-18

**Authors:** Moa Pontén, Oskar Flygare, Martin Bellander, Moa Karemyr, Jannike Nilbrink, Clara Hellner, Olivia Ojala, Johan Bjureberg

**Affiliations:** Centre for Psychiatry Research, Department of Clinical Neuroscience, Stockholm, Karolinska Institutet, Sweden & Stockholm Health Care Services, Region Stockholm, Norra Stationsgatan 69, 113 64 Stockholm, Sweden

**Keywords:** Artificial intelligence, Random forest analysis, Nonsuicidal self-injury, Emotion regulation, Machine learning

## Abstract

**Background:**

Nonsuicidal self-injury is a common health problem in adolescents and associated with future suicidal behavior. Predicting who will benefit from treatment is an urgent and a critical first step towards personalized treatment approaches. Machine-learning algorithms have been proposed as techniques that might outperform clinicians’ judgment. The aim of this study was to explore clinician predictions of which adolescents would abstain from nonsuicidal self-injury after treatment as well as how these predictions match machine-learning algorithm predictions.

**Methods:**

Data from a recent trial evaluating an internet-delivered emotion regulation therapy for adolescents with nonsuicidal self-injury was used. Clinician predictions of which patients would abstain from nonsuicidal self-injury (measured using the youth version of Deliberate Self-harm Inventory) were compared to a random forest model trained on the same available data from baseline assessments.

**Results:**

Both clinician (accuracy = 0.63) and model-based (accuracy = 0.67) predictions achieved significantly better accuracy than a model that classified all patients as reaching NSSI remission (accuracy = 0.49 [95% CI 0.41 to 0.58]), however there was no statistically significant difference between them. Adding clinician predictions to the random forest model did not improve accuracy. Emotion dysregulation was identified as the most important predictor of nonsuicidal self-injury absence.

**Conclusions:**

Preliminary findings indicate comparable prediction accuracy between clinicians and a machine-learning algorithm in the psychological treatment of nonsuicidal self-injury in youth. As both prediction approaches achieved modest accuracy, the current results indicate the need for further research to enhance the predictive power of machine-learning algorithms. Machine learning model indicated that emotion dysregulation may be of importance in treatment planning, information that was not available from clinician predictions.

**Trial Registration:**

NCT03353961||https://www.clinicaltrials.gov/, registered 2017–11-21.

Preregistration at Open Science Framework: https://osf.io/vym96/.

**Supplementary Information:**

The online version contains supplementary material available at 10.1186/s12888-024-06391-x.

## Introduction

Nonsuicidal self-injury (NSSI) is a common health problem in adolescents [1]. Possible adverse outcomes associated with NSSI include suicidal behavior, thus making NSSI an important treatment target [2]. Non-suicidal self-injury disorder (NSSID) was proposed as a diagnostic category in the Fifth Edition of the Diagnostic and Statistical Manual of Mental Disorders (DSM-5). To meet the diagnostic criteria, the following criteria need to be fulfilled: (1) engagement in NSSI on 5 or more days in the past year (Criterion A); (2) the expectation that NSSI will solve an interpersonal problem, provide relief from unpleasant thoughts and/or emotions, or induce a positive emotional state (Criterion B); (3) the experience of one or more of the following: (a) interpersonal problems or negative thoughts or emotions immediately prior to NSSI, (b) preoccupation with NSSI that is difficult to manage, or (c) frequent thoughts about NSSI (Criterion C); (4) the NSSI is not socially sanctioned or restricted to minor self-injurious behaviors (Criterion D); (5) the presence of NSSI-related clinically significant distress or interference across different domains of functioning (e.g., work, relationships; Criterion E); and (6) the NSSI does not occur only in the context of psychosis, delirium, or substance use/withdrawal and is not better accounted for by another psychiatric disorder or medical condition (Criterion F) [3].

Treatment of NSSI is often time-consuming and difficult to access [4]. Novel treatment approaches, such as Internet-delivered Emotion Regulation Individual Therapy (IERITA [5]), are developed to make treatment more accessible for this population. Given the heterogeneity across adolescents with NSSI [1], understanding which patients benefit from different therapies is an essential objective in a personalized treatment approach. This approach may help clinicians identify patients who could benefit from more intensive or supplementary interventions if a poor treatment outcome is predicted.

Clinical work involves decision-making, ranging from micro-level choices during a patient visit to macro-level predictions necessary for treatment triangulation based on clinical guidelines and patients’ characteristics. By combining clinical experience with the clinical presentation of the individual’s symptoms and history, the clinician forms an intuitive “gut-feeling” whether the treatment will be effective [6]. This clinical judgement is often preferred by the clinicians themselves [6]. However, clinical prediction tends to overestimate treatment effect, failing to identify individuals at risk for worse treatment outcome [7]. Further, experience, training and/or consultation seem to only marginally improve clinical judgement [8]. With the goal to improve prediction, statistical methods have been explored as an alternative or complement to clinical judgement [9–11].

Machine learning (ML) is a set of techniques that are promising in predicting disease and treatment outcome [12] and marks a paradigm shift also in psychiatry research [13]. These statistical tools allow multiple variables to be examined simultaneously, even correlated ones, and can illustrate complex non-linear patterns. Historically, research has focused on single variables, such as clinical characteristics, genes or brain data in predicting treatment outcome. However, none of these predictors alone have shown a large effect in psychiatric research. Therefore, machine learning methods are exceptionally well suited for predicting treatment outcomes as they allow aggregating small effects. One example of clinical application is from a trial for body dysmorphic disorder where the treatment outcome was predicted with 78% accuracy, indicating potential for clinical utility for these methods [14].

Although ML increasingly has been used to predict treatment outcome in psychiatric research [13], only a few studies have compared ML to clinicians in predicting treatment outcome. In two studies on psychological treatment for alcohol dependence the authors found that the ML is comparable to clinical judgment [15] and that ML outperformed clinicians’ intuition [11].

Based on data from a randomized clinical trial conducted by our group [16], this study sought to explore the accuracy of clinician prediction of treatment response following IERITA, an internet-delivered intervention for adolescents with NSSID. We also wanted to explore the difference between a machine learning (ML) algorithm compared to clinicians in predicting treatment outcomes and explore the most important predictors. As an exploratory model development study, we did not form specific a priori hypotheses regarding predictors.

Given the limited literature on what informs and impacts clinician prediction of outcomes we wanted to explore the following secondary aims (1) if clinician confidence was associated with accuracy in prediction; (2) if the confidence and/or accuracy of clinician predictions improved as therapist treated more patients in the trial; (3) if clinician predictions were related to the amount of time they spent on treatment and how many messages they sent to the patient; and finally (4) if clinician predictions of NSSI outcome were related to the number of treatment modules the patient completed.

## Materials and methods

### Design

Data was gathered from a recent randomized clinical trial (*N* = 166) conducted at three sites within Child and Adolescent Mental Health Services in Sweden (NCT03353961||https://www.clinicaltrials.gov/,registered 2017–11–21) where the intervention was superior to the treatment as usual (TAU) control condition in reducing nonsuicidal self-injury [17]. Those randomized to TAU were offered IERITA after six months. Recruitment took place between November 20, 2017, to April 9, 2020 with follow-up January 2021. The trial was approved by the Stockholm Regional Ethical Review Board (no. 2017/1807–31), and all participants provided written informed consent, with older participants providing written consent and younger participants verbal consent with parental written consent. Random allocation sequence was conducted by an independent researcher using a true random number service (Random.org) in blocks of 4 or 6 for each treatment clinic and stored in sealed, opaque envelopes. The Consolidated Standards of Reporting Trials (CONSORT) and TRIPOD reporting guidelines were followed in the reporting of this study [18].

### Participants

**Fig. 1:**
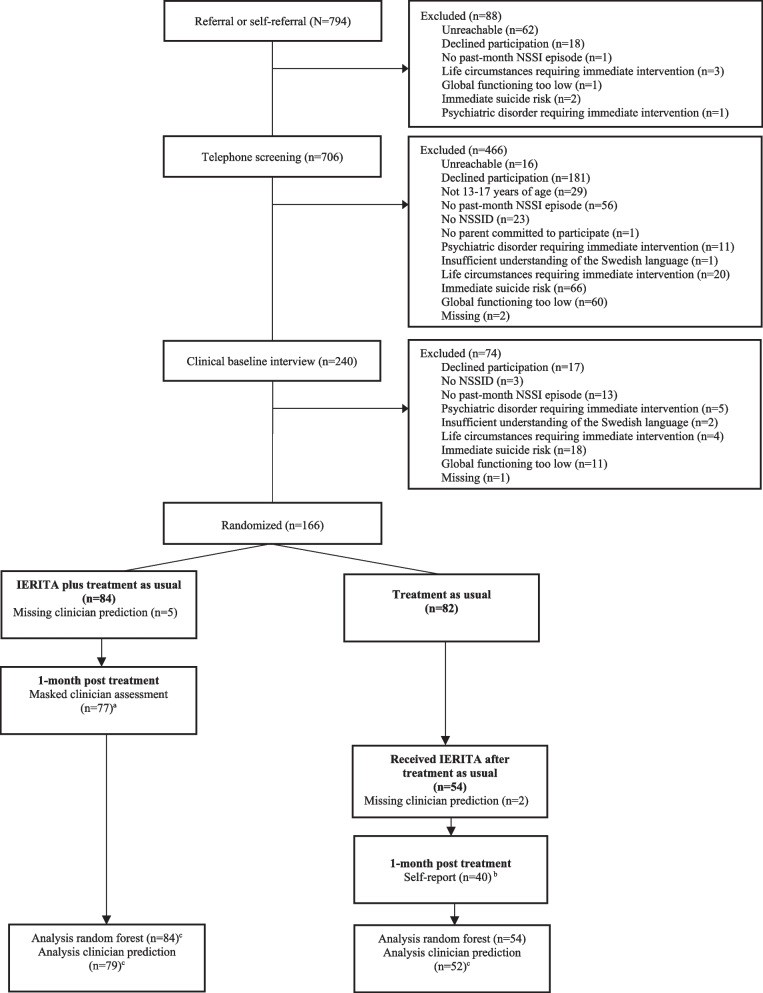
Flow Diagram of Patient Enrollment and Disposition. IERITA indicates internet-delivered emotion regulation individual therapy for adolescents; NSSID, nonsuicidal self-injury disorder.^a^ Masked Assessor-rated Deliberate Self-Harm Inventory–Youth Version. ^b^ Self-reported Deliberate Self-Harm Inventory–Youth Version. ^c^ All randomized participants for which a clinician prediction was made are included in the analysis

The current study included participants (*n* = 138) across groups, i.e. those who received Internet-delivered Emotion Regulation Individual Therapy (IERITA) immediately (*n* = 84) and those assigned to TAU who later enrolled in IERITA (*n* = 54) (see Fig. 1). Both groups received treatment for 12 weeks. For baseline demographics such as NSSI frequency see Table 1. No a priori power analysis was made since the study was based on already collected data. Inclusion criteria comprised adolescents aged 13 to 17 with NSSI disorder and experiencing ≥ 1 NSSI episode during the past month (measured using the youth version of Deliberate Self-harm Inventory), with one parent willing to join the parent program [17]. Exclusion criteria involved a Children’s Global Assessment Scale (CGAS) score below 40 [19], insufficient understanding of the Swedish language, immediate suicide risk, psychotic or bipolar I disorder diagnosis, current (past month) substance use disorder, life circumstances hindering participation, or other psychiatric disorder requiring immediate treatment [17].

**Table 1:** Participant Characteristics

	**No. (%)**
	**Total** **(*n* = 138)**
**Participant Characteristics**	
Gender	
Female	126 (91)
Male	7 (5)
Non-binary	5 (4)
Age, mean (SD)	15.03 (1.24)
Sexual orientation	
Heterosexual	92 (67)
Sexual minority	43 (31)
No answer	3 (2)
Any failed grades (yes)	19 (14)
Ever been bullied (yes)	71 (51)
School absence, mean (SD)	4.39 (4.97)
**Parent Characteristics**^a^	
Parent living arrangement	
With children	112 (81)
With spouse/partner	101 (73)
Parent education level	
Primary school	2 (1)
Secondary school	54 (39)
College/university < 3 years	13 (9)
College/university ≥ 3 years	60 (43)
Doctorate	9 (7)
Emotion dysregulation, mean (SD)	29.15 (10.85)
Parental ability (Coping with Children's Negative Emotions Scale)	
Problem-Focused, mean (SD)	5.77 (0.83)
Emotion-Focused, mean (SD)	5.1 (0.95)
Expressive Encouragement, mean (SD)	5.24 (0.94)
Minimization, mean (SD)	2.8 (1.02)
Punitive, mean (SD)	1.49 (0.54)
Distress, mean (SD)	1.84 (0.86)
	**No. (%)**
	**Total** **(*n* = 138)**
**Participant Clinical Characteristics**	
Age NSSI onset, mean (SD),	12.67 (1.38)
Years since NSSI onset, mean (SD)	2.36 (1.31)
NSSI frequency in the past 30 days, DSHI-Y, mean (SD)	3.07 (3.54)
NSSI versatility, DSHI-Y, mean (SD)	1.30 (1.02)
Comorbidity^b^	
Major depressive disorder	77 (56)
Dysthymia^e^	6 (4)
Anxiety disorders	
Social anxiety disorder	41 (30)
Panic disorder	12 (9)
Agoraphobia	18 (13)
Specific phobia disorder	23 (17)
Generalized anxiety disorder	19 (14)
Separation anxiety^e^	3 (2)
OCD^e^	4 (3)
BDD^e^	6 (4)
ADHD^c^	26 (19)
Autism spectrum disorder^e^	5 (4)
Anorexia^e^	1 (1)
Bulimia^e^	6 (4)
Oppositional defiant disorder^e^	4 (3)
Depression, Anxiety and Stress Scale (DASS-21)	
Depression, mean (SD)	12.09 (4.83)
Anxious, mean (SD)	8.25 (4.19)
Stress, mean (SD)	11.54 (4.36)
Insomnia symptoms, ISI, mean (SD)	11.18 (5.62)
Number of co-occurring disorders, mean (SD)	1.82 (1.57)
Number of BPD criteria^d^, mean (SD)	1.93 (1.35)
Self-destructive behaviours, BSL, mean (SD)	2.8 (2.46)
Suicidality	
Low	57 (41)
Moderate	36 (26)
High	45 (33)
Life-time suicide attempt, yes	19 (14)
Ever received inpatient care, yes^e^	3 (2)
Emotion dysregulation, Ders-16, mean (SD)	58.50 (12.12)
Quality of life, Kid-Screen, mean (SD)	28.58 (4.38)
Psychological flexibility, AAQ, mean (SD)	31.68 (8.18)
Any ongoing psychopharmacological medication, mean (SD)	50 (36)
Time in ongoing counselling, mean (SD), mo	9.67 (12.36)
Ongoing counselling at inclusion	97 (70)
Global functioning, (CGAS) mean (SD)	54.27 (5.84)
Clinical severity (CGI-S) mean (SD)	
Mildly ill (3)	24 (17)
Moderately ill (4)	73 (53)
Markedly ill (5)	36 (26)
Severely ill (6)	5 (4)

*Abbreviations: AAQ* Action and Acceptance Questionnarie, *ADHD* attention-deficit hyperactivity disorder, *BDD* body dysmorphic disorder, *BSL* Borderline Symptom List, *BPD* borderline personality disorder, *CGI-S* Clinical global impression-severity, *CGAS* Children’s Global Assessment Scale, *DERS* The Difficulties in Emotion Regulation Scale, *DSHI-Y* Deliberate Self-Harm Inventory–Youth Version, *ISI* Insomnia severity index, *NSSI* nonsuicidal self-injury, *OCD* obsessive–compulsive disorder

^a^In case of two parents, one was assigned and consented to contribute to answer self-reports questions. Multiple answers were allowed

^b^Assessed by the research team using the MINI-KID International Neuropsychiatric Interview, version 6 and the Body Dysmorphic Disorder Questionnaire (administered as an interview)

^c^Includes both combined, primarily inattentive, and primarily hyperactive-impulsive subtype

^d^Assessed by the research team using the Structured Clinical Interview for DSM

^e^Not included in the final analysis due to insufficient variance

### Interventions

IERITA is a clinician-supported 12-week acceptance-based behavioral therapy, aiming to reduce NSSI by improving emotion regulation ability. Adolescents receive 11 weekly modules, incorporating text, films, and interactive exercises to learn emotion regulation skills. A supplementary mobile app facilitates learning and skill practice. A detailed description of IERITA is found in Bjureberg et al., 2023 [17].

IERITA was offered as an addition to TAU. TAU was delivered within regular healthcare services according to needs (e.g., pharmacological treatment or supportive therapy), resulting in varying types and frequencies of treatments for the participating adolescents outside the trial. The TAU only condition was enhanced by referral to adequate treatment if necessary, the establishment of a safety-plan, self-rated assessments every week, and follow-up assessments.

During IERITA, patients had asynchronous contact via a message function in the platform with a dedicated clinician who reinforced treatment engagement and assisted with homework assignment by giving corrective feedback and psychoeducation. The clinicians (*n* = 15) were either psychologists or psychotherapists and the vast majority worked within child and adolescent healthcare. The clinicians received structured training and support, emphasizing adherence to the protocol.

### Measures

The outcome of interest was the proportion of patients with an absence of NSSI (yes/no) at one-month post-treatment, as reported in a youth version of Deliberate Self-harm Inventory (DSHI-Y; [20, 21]), which has shown adequate construct, convergent, and discriminant validity [20]. DSHI-Y measures the frequency of the 6 most common forms of NSSI (eg, cutting and burning in the past 30 days) without conscious suicidal intent, but resulting in injury severe enough for tissue damage (e.g., scarring) to occur. For the adolescents receiving IERITA straight after randomization, DSHI-Y was assessed by a clinician blind to allocation and independent from the scientific team. For the adolescents receiving IERITA six months later, DSHI-Y was self-reported one month post-treatment. The outcome was measured with reference to the 30 days post-intervention in both groups.

### Predictors

Clinicians predicted adolescent NSSI abstinence with a yes/no response and rated confidence in percentages in steps of 10 (i.e., 0, 10 […] or 100%) after a face-to-face assessment but prior to randomization. There was one prediction made per patient. Demographic and clinical variables measured at baseline were available to clinicians and used in the ML model (Table 1). Predictor variables in the ML model were selected to match the information available to clinicians. Clinicians did not receive specific instructions on what information to use in their predictions or how to weigh predictors but were instructed to use their “clinical hunch”.

### Statistical analyses

#### Preprocessing and feature selection

For the machine learning model a random forest model was used [22]. The data was pre-processed by keeping predictors that had sufficient variance and were independent from other predictor variables and had no more than 30% missing data, in line with previous studies in this field [14, 23, 24]. Missing data in all predictors were imputed using bagged trees using the *tidymodels* R-package [25, 26].

For the primary outcome variable (21 missing observations), missing data was imputed using predictive mean matching based on the weekly ratings of NSSI episodes collected during treatment [27], excluding the predictor variables to prevent leakage [28]. The random forest model was fitted without hyperparameter tuning using the *ranger* package and with internal validation in order to reduce the risk of overfitting; i.e. always growing 500 trees, selecting the square root of the total number of predictors at each split, using a minimum node size of 10, and using tenfold cross-validation [29]. Variable importance was estimated using corrected Gini importance [30] and corresponding permutation-based *p*-values were estimated [31].

The random forest model and clinician predictions were compared using accuracy (the proportion of correct predictions), sensitivity/specificity (the proportion of patients with/without absence of NSSI correctly detected), positive predictive value/negative predictive value (the proportion of true positives/negatives among the model predictions) as well as receiver operating characteristics (ROC) curves and their corresponding area under the curve (AUC). Further, we applied McNemar’s test to evaluate whether there was a statistically significant difference in classification performance between clinician and random forest predictions [32].

The association between clinician’s confidence (0–100%) and the accuracy of clinician predictions was tested using a logistic mixed-effect model with random intercept, with accuracy (0/1) as the dependent variable and confidence as the independent variable (*n* = 130). To investigate if clinician accuracy improved over time a logistic mixed-effects model with random intercept was employed, with prediction accuracy as the dependent variable, and the patient order, i.e. the number of patients the clinician had treated as the independent variable (*n* = 130). The clinician confidence over time was tested using a mixed-effects model with random intercept, with prediction confidence as the dependent variable, and the patient order as the independent variable (*n* = 130). Whether therapist predicted probabilities related to time spent treating (*n* = 61) and number of messages sent (*n* = 63) was tested using a mixed-effects model with random intercept for each of the two dependent variables (time spent, number of messages sent) and predicted probability as the independent variable. Patients were only included in this analysis if the therapist making the prediction were also treating the patient. The relationship between therapist predictions and number of modules completed (treatment dose) was investigated using a linear regression, with number of completed modules as the dependent variable and predicted probability and baseline NSSI as the independent variables (*n* = 79).

All statistical analyses were performed using R version 4.3.1 [33]. The pre-registered statistical analysis plan, as well as scripts used to produce the results, are available on the Open Science Framework (https://osf.io/vym96/). We originally planned in secondary analyses to evaluate whether patients would improve, deteriorate, or have no change on the CGI-I (Clinical Global Impression scale–Improvement) in addition to absence of NSSI, however these analyses were not feasible as clinicians predicted that 98% of all patients would improve after treatment and CGI-I data were only available for 80 patients.

## Results

The random forest model utilized data from all participants (*n* = 138), however clinician predictions were unavailable for 7 patients and clinician predictions are therefore based on estimates from *n* = 131 participants.

Clinicians predicted NSSI absence for 80 (58%) patients, NSSI presence for 51 (37%) patients and prediction was missing for 7 (5%) patients. During the post-treatment follow-up period 60 (44%) patients did not engage in self-harm, 57 (41%) patients did engage in self-harm, while data was missing for 21 (15%) patients. The confidence ratings by the clinicians ranged from 0 to 100 (mean = 57.3, SD = 18.1).

### Clinician predictions

**Fig. 2:**
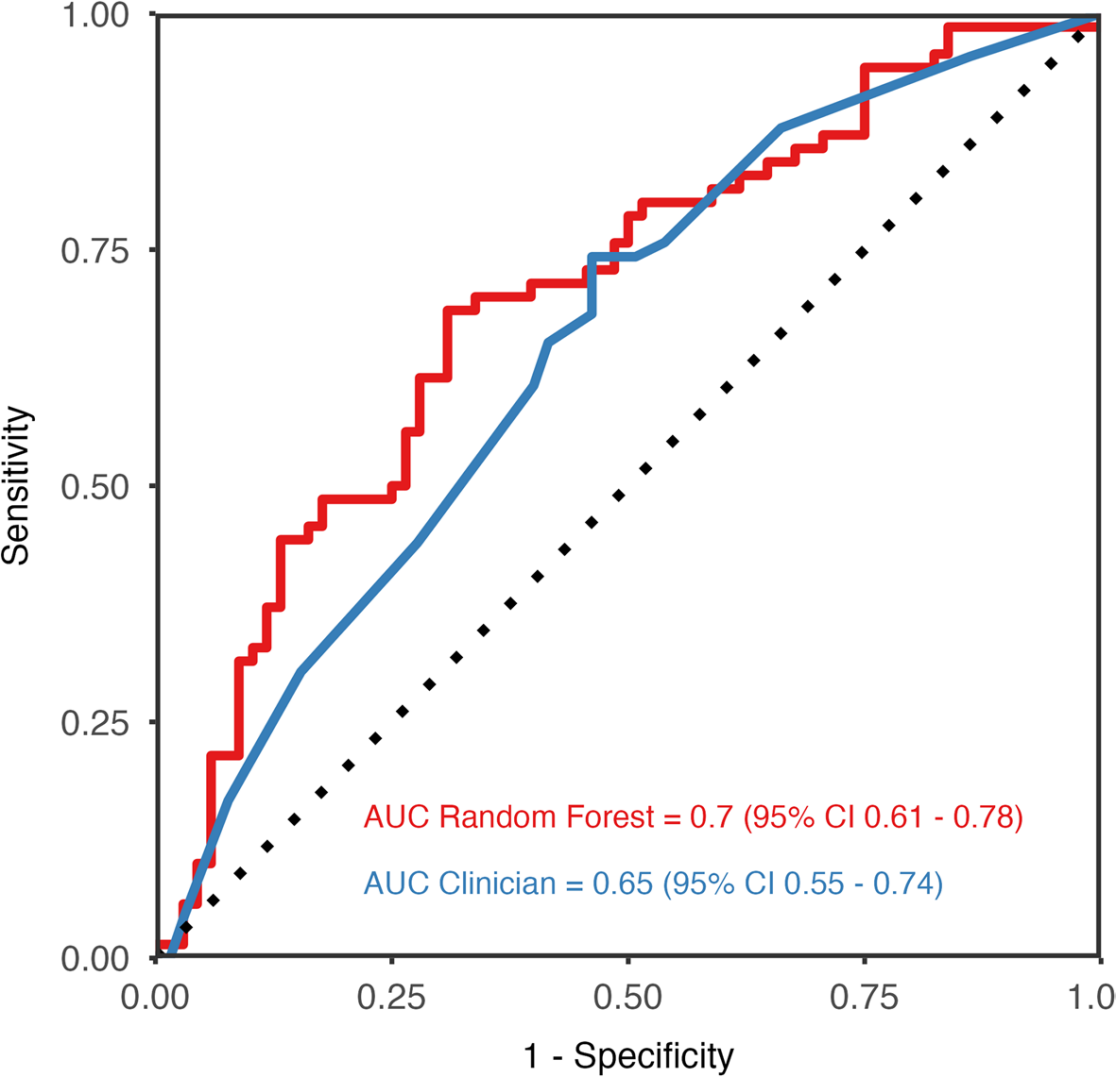
Receiver operating characteristics curves for clinician and ML predictions**.** *Abbreviation:* AUC, area under the curve

Area under the curve for the clinician predictions was 0.65 (95% CI 0.55 to 0.74) (see Fig. 2). The clinician predictions achieved an overall accuracy of 0.63, a sensitivity of 0.74 and specificity of 0.52. The positive predictive value was 0.61 and the negative predictive value was 0.67.

### Machine learning predictions

During pre-processing, information regarding previous inpatient admissions and nine rare comorbid conditions were removed due to near-zero variance. No predictor variables were removed due to high collinearity or large proportion of missing values, and the final random forest algorithm ended up using 44 predictor variables (listed in Table 1).

Area under the curve for the random forest model was 0.7 (95% CI 0.61 to 0.78) (see Fig. 2). The model reached an accuracy of 0.67, with a sensitivity of 0.61 and specificity of 0.72. The positive predictive value was 0.69 and the negative predictive value was 0.64.

**Fig. 3:**
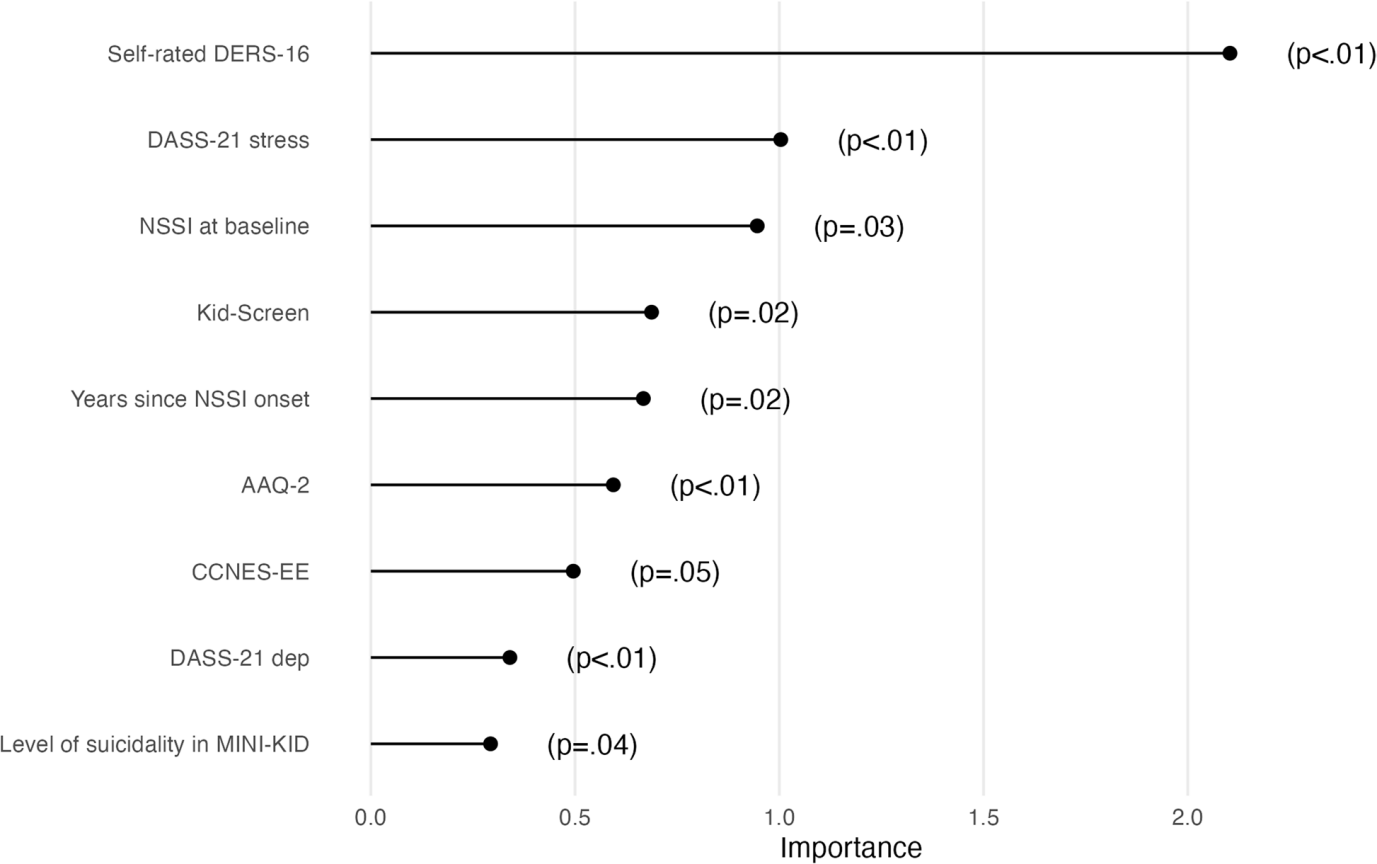
Predictor importance of top 9 variables identified by the algorithm predicting self-injury post-treatment. The most important predictor was self-rated emotion dysregulation on the DERS-16 at baseline (Importance = 2.10, *p* < .01), followed by self-rated stress symptoms in DASS-21 (Importance = 1.0, *p* < .01), number of NSSI episodes on the DSHI-Y at baseline (Importance = 0.95, *p* = .029), quality of life on the Kid-Screen (Importance = 0.67, *p* = .019), number of years since NSSI onset (Importance = 0.67, *p* = .019), psychological flexibility according to AAQ-2 (Importance = 0.59, *p* < .01), parental expressive encouragement on the CCNES (Importance = 0.50, *p* = .049), self-rated depression symptoms in DASS-21 (Importance = 0.34, *p* < .01), level of suicidality in MINI-KID (Importance = 0.29, *p* = .039). Abbreviation: DERS-16, Difficulties in Emotion Regulation Scale; CCNES-EE, Coping with Children's Negative Emotions Scale, Expressive Encouragement; DASS-21, Depression, Anxiety and Stress Scale – 21; NSSI, nonsuicidal self-injury; AAQ-2, Action and Acceptance Questionnaire

The 9 most important predictors (*p*-value < 0.05) in the random forest model are shown in Fig. 3, where difficulties in emotion regulation were identified as the most important predictor of nonsuicidal self-injury absence after treatment.

### Clinician predictions vs machine learning

Both the random forest model and the clinician predictions were more accurate than a baseline model predicting that all patients would reach NSSI absence, which had an accuracy of 0.49 (95% CI 0.41 to 0.58). The random forest model and clinician predictions did not differ in terms of accuracy (0.67 and 0.63 respectively, McNemar test = 0.205, df = 1, *p* = 0.65), and adding the clinician prediction to the random forest model did not improve accuracy compared to the random forest model alone (Accuracy = 0.65, McNemar test = 0.308, df = 1, *p* = 0.58). Both predictions were correct for 37 (28%) patients, and there was agreement between therapists and the random forest model for 57 (44%) patients.

### Secondary aims

We did not find evidence for an association between clinicians’ confidence and the accuracy of their predictions (β = 0.012, SE = 0.011, *p* = 0.269). Furthermore, we failed to find evidence that clinicians’ accuracy or confidence improve with number of patients they predicted and treated, since the order of patient was not associated with either clinicians’ accuracy of prediction (β = 0.017, SE = 0.032, *p* = 0.601) or clinicians’ confidence in their prediction (β = 0.156, SE = 0.243, *p* = 0.523). Therapist spent on average 241 min (SD = 221) treating each patient and sent on average 16.4 messages (SD = 7.22). The clinicians’ prediction probabilities were not associated with the time spent on treatment (β = −11.033, SE = 93.645, *p* = 0.907) or the number of messages they sent (β = −1.913, SE = 2.532, *p* = 0.453). Lastly, the number of modules completed by patients was not associated with clinicians’ prediction probabilities (β = −0.482, SE = 0.876, *p* = 0.584, *n* = 79).

### Sensitivity analyses

To evaluate whether the method for assessing NSSI absence (clinician-rated or self-rated) impacted model accuracy, we compared model performance for patients receiving IERITA immediately and patients receiving IERITA after TAU. The accuracy was slightly higher in the first group (accuracy = 0.70, sensitivity = 0.67, specificity = 0.74) compared to the latter (accuracy = 0.61, sensitivity = 0.52, specificity = 0.69), however the confidence intervals of the area under the curve metric overlapped (see eFigure 1 in supplemental materials, McNemar test not possible due to differing number of participants in the groups).

## Discussion

This study explored the predictive abilities of clinicians and a random forest model for treatment response following an internet-delivered emotion regulation treatment for adolescents with NSSI. Findings suggest that both clinicians and the ML algorithm performed above chance but at comparable accuracy rates (63% for clinicians and 67% for ML). However, these accuracies do not meet current thresholds for clinical application. Clinician confidence was not associated with accuracy in prediction, and neither clinician confidence nor accuracy improved over time. Therapist predictions and confidence ratings showed no association with therapist time spent on asynchronous contact with individual patients, including the number of messages sent and time spent per patient.

This study adds to the limited literature on mental health-clinicians vs ML predictions of post-treatment outcome. In contrast with previous findings [11], our data suggest that ML and clinician predictions are comparable. However, although better than chance, prediction accuracies of 63% (clinicians) and 67% (ML) are not useful or ready for implementation in clinical practice. There is evidence that clinicians view predictions with at least 65% accuracy as suitable for practical application [34], and recent empirical findings suggest that an accuracy of 67% should be a minimum benchmark for clinically useful predictions [35].

Importantly, the ML prediction did not improve after including the clinician prediction, suggesting that clinical intuition does not add information above and beyond the observable data available to the ML. Thus, even though clinicians interacted with patients face-to-face at baseline and had the opportunity to form clinical assumptions based on the patient's behavior during this interaction, this information did not appear to be crucial for prediction; at least in situations when rich baseline data is available. Instead, our results suggest that the information associated with clinical intuition, that was a statistically significant predictor of NSSI absence, may be observable in self-report data. Future research should delineate what information clinicians use when making clinical predictions, eg. when tailoring a treatment to an individual patient’s characteristics. Additionally, this may include asking them to rank the relative importance of these factors to see similarities and differences to those factors that emerged from the random forest model.

It is noteworthy that clinician predictions were based on intuitive information from both face-to-face patient interactions and observable baseline patient data, the same information typically employed in regular care, thereby enhancing the study’s ecological validity. This is in contrast to studies with clinicians only examining baseline patient data before making their prediction [15, 36].

Further, the clinicians’ accuracy in prediction did not improve with time after meeting more patients. This aligns with previous data suggesting that clinical predictions may not be useful in clinical practice across a range of disorders and treatment modalities [7, 11]. This may not be surprising given humans’ limited capacity to process and weigh information based on more than four variables [37]. Our preliminary findings suggest that ML is already on par with clinicians’ ability to predict outcome and future research should focus on identifying key variables that may further improve the prediction accuracy of ML.

Entering the trial with high levels of emotion dysregulation emerged as the most important predictor in the ML, information that was not available from the clinician predictions. These findings indicate that a machine learning model may add information beyond clinician prediction and that patients with more severe difficulties with emotion regulation are more likely to abstain from NSSI the month after this brief internet-delivered intervention. This aligns with prior findings demonstrating a positive association between clinical severity and treatment response in treatments targeting emotion dysregulation in patients with NSSI (e.g., [38–41]), potentially partly explained by regression to the mean, where high levels of a symptom may normalize over time. In addition, it is possible that self-harm that is not driven by emotion dysregulation may be less responsive to this particular treatment. Furthermore, it is important to note that the effect of one predictor is impacted by all other predictors in the ML model. We have previously found that emotion dysregulation and NSSI did not strongly predict treatment outcome when investigated separately in simple regression analyses [16]. Further, we have recent findings indicating that it might be particular patterns of week-to-week variability in baseline emotion dysregulation that is associated with treatment outcome [42]. Future research should delineate the predictive role of emotion dysregulation by studying how it interacts with other variables and varies over time before and after enrolling treatment.

Finally, previous research has shown that clinicians often overestimate patients improvement and ML algorithms are better at predicting those who deteriorate [11]. Predictions based on ML can therefore complement clinician predictions, as it is clinically more relevant to identify those in need of more resources. The PPV, or the likelihood that a positive prediction indicates a true need for intervention, was 69% for ML and 61% for clinician prediction. The NPV, or the likelihood that a negative prediction accurately indicates a lack of need for intervention, was 64% for ML and 67% for clinician prediction. Given that IERITA is brief and low resource intensive [17] there is little room for reducing intervention dose based on a strong prognosis. Future research might therefore consider weighing PPV higher than NPV in the algorithm, as it may be more critical to identify those truly in need of additional support. This approach has for example been shown to lead to improved outcomes in the treatment of insomnia in adults [35].

### Limitations

First, the relatively limited sample size did not allow us to separate some of the data for testing, a practice preferably undertaken in ML [25, 43] and it is currently unknown if the observed results will generalize to new samples. The limited sample size also increased the risk of overfitting, which remains a challenge despite only selecting variables available to clinicians and avoiding hyperparameter tuning of the random forest model. The utility of ML in predicting treatment outcomes for adolescents with NSSI will require future research using larger more varied samples. Second, although the current study employed a wide range of different variables in the ML algorithm, including clinical, parent-reported and sociodemographic variables, as well as contextual factors such as school engagement and bullying, future studies should explore how the algorithm can be optimized by for example including ecological momentary assessment data [44] and semi-objective data such as pain measures [45], paying particular attention to minimizing measurement error [46]. Third, the outcome assessed in this study was absence of self-injury. However, there are other possible outcomes of interest, for example CGI, which we were not able to assess in the current study. Fourth, the method to determine NSSI absence differed between the group receiving IERITA immediately (clinician-rated) and the group receiving after six months (self-rated). However, exploratory analysis showed no statistically significant difference in the random forests’ performance when predicting NSSI absence in the two groups. Fifth, this study included only one treatment (IERITA), limiting the generalizability to research investigating treatment selection based on patients’ predicted response. Finally, the generalizability is limited by the predominately female sample.

## Conclusions

This study showed that clinicians were better than chance in predicting NSSI absence at post-treatment. The model development part of the study highlights the potential for ML models to match clinician predictions of treatment response for adolescents with NSSI and identified emotion dysregulation as the most important predictor in the ML model. Given that both prediction methods achieved only modest accuracy, these findings suggest the need for further research to deepen our understanding of treatment response prediction in this population.

## Supplementary Information

Supplementary Material 1.

## Data Availability

The data used for analyses contain sensitive personal identifying information and are not publicly available as data sharing was not part of the written informed consent. Data are available from the corresponding author on reasonable request. Statistical code used for the analyses is publicly available from the Open Science Framework repository: https://osf.io/vym96/).
