# Supplementary material for: Comparison between clinician and machine learning prediction in a randomized controlled trial for nonsuicidal self-injury
Source: BMC Psychiatry. 2024 Dec 18;24:904. doi: 10.1186/s12888-024-06391-x (PMC11653784; doi:10.1186/s12888-024-06391-x)
Supplement: Supplementary file 1 — Supplementary Material 1. [file 12888_2024_6391_MOESM1_ESM.pdf]

SUPPLEMENTAL ONLINE CONTENT

Comparison between clinician and machine learning prediction in a randomized controlled trial for nonsuicidal self-injury

Moa Pontén*, PhD, Oskar Flygare, PhD, Martin Bellander, PhD, Moa Karemyr, MSc, Jannike Nilbrink, MSc, Clara Hellner, PhD, Olivia Ojala, PhD, Johan Bjureberg, PhD

Measures

Diagnostic assessments

A full diagnostic interview was conducted using the MINI-KID International Neuropsychiatric Interview, version 6 (1) and Body Dysmorphic Disorder Questionnaire (administered as an interview;(2). The Clinician-Administered Nonsuicidal Self-Injury Disorder Index(3) including items corresponding to the Deliberate Self Harm Inventory (DSHI-Y)(4,5) was used to determine if participants met criteria for NSSID, and the Structured Clinical Interview for DSM-IV Personality Disorders Borderline Personality Disorder (BPD) Module was used to assess diagnostic criteria for BPD(6). The CGI-S was conducted to evaluate the severity of psychopathology(7). CGI-S is a one-item measure (*"Considering your total clinical experience with this particular population, how mentally ill is the patient at this time?"*) ranging from 1 to 7, where 1 is 'normal' and 7 is 'among the most extremely ill patients'.

Self-Rated Measures

NSSI frequency was measured by the youth version of the Deliberate Self Harm Inventory (DSHI-Y)(4,5). The DSHI-Y assesses the presence and frequency of the 6 most common forms of NSSI, including cutting, burning, severe scratching, self-biting, self-punching, and head banging.

Emotion dysregulation was assessed using the 36-item Difficulties in Emotion Regulation Scale (DERS)(8). Scores on this measure range from 36 to 180, with higher scores indicating greater

emotion dysregulation. The DERS has demonstrated good reliability and construct and convergent validity in adolescents(9,10). Internal consistency in this sample was excellent (α = .90).

Emotion dysregulation was also measured using the 16-item version of the DERS, the DERS-16 (11). Scores on this measure range from 16 to 80, with higher scores indicating greater emotion dysregulation. The DERS-16 has been found to demonstrate good test-retest reliability among adults(11), as well as good reliability and validity and measurement and structural invariance across age among adolescents(9). Internal consistency in this sample was excellent (α = .92).

Past week engagement in a variety of risky, self-destructive behaviours (e.g., suicide attempts, risky sexual behaviour, binge eating, substance misuse) was measured using the 11-item behaviour supplement to the Borderline Symptom List (BSL)(12). Scores on this measure range from 0 to 55. At the end of BSL during weeks 1 to 16, we also added a question regarding suicidal ideation during the past week, where participants could choose any of the following answers: "*I am not thinking about killing myself*", "*I am thinking about killing myself, but would never do it*", and "*I want to kill myself*".

The DASS-21(13) is a 21-item self-report measure of depression, anxiety, and stress symptoms experienced in the past week. Psychometric studies suggest that it is best represented by a total scale score as an overall marker of psychiatric symptoms (14) Items are answered on a 4-point Likert-type scale, with higher scores indicating more severe symptoms. The DASS-21 has demonstrated good validity and reliability(14), and its total scale score had good internal consistency in our sample (α = .89).

The self-reported Insomnia Severity Index (ISI) was used to assess insomnia. Total scores range from 0 to 28 (15). The suggested cutoff score for clinical insomnia in youths is ≥9(16).

The Acceptance and Action Questionnaire (AAQ-II; (17)) is a 7-item measure of psychological inflexibility/experiential avoidance. Response options range from 1 (never true) to 7 (always true), and higher scores indicate greater inflexibility. An example AAQ-II item is "I'm afraid of my feelings." The AAQ-II has been found to have good internal consistency (α = 0.84) and good test-retest reliability at 3 months (0.81) and at 12 months (0.79).

The Kid-Screen 10 is a short self-rated assessment of health-related quality of life that was administered at pre-treatment, 1-month post-treatment and 3-month post-treatment. The questionnaire measures the young person's subjective health and quality of life in the domains of physical and mental well-being, independence, school environment, and relationship to parents and friends (18). Kid-Screen 10 has adequate internal consistency (α = .81)(19).

Parents' perceived ability to handle and respond to their children's negative emotions was evaluated using the Coping with Children's Negative Emotions Scale (CCNES). (20) The CCNES-A presents parents with nine hypothetical scenarios that depict typical situations likely to evoke teenagers' negative emotions (e.g., "W*hen I see my teenager become anxious about something at school, I usually*"). Parents are asked to rate the likelihood of using different responses to their child's negative emotions on a seven-point Likert-type scale. The total subscale score ranges from 1-7. Lower scores indicate more desired response, except for the subscale expressive encouragement, for which higher scores indicate more desired response.(20)

Masked Assessor-Rated Measures

Global functioning was assessed using the Children's Global Assessment Scale (CGAS)(21). Scores on this measure range from 0-100, with higher scores indicating better functioning. CGAS has shown moderate to excellent inter-rater reliability, good stability over time, and good concurrent and discriminant validity has been provided (21,22).

Results

Sensitivity analyses

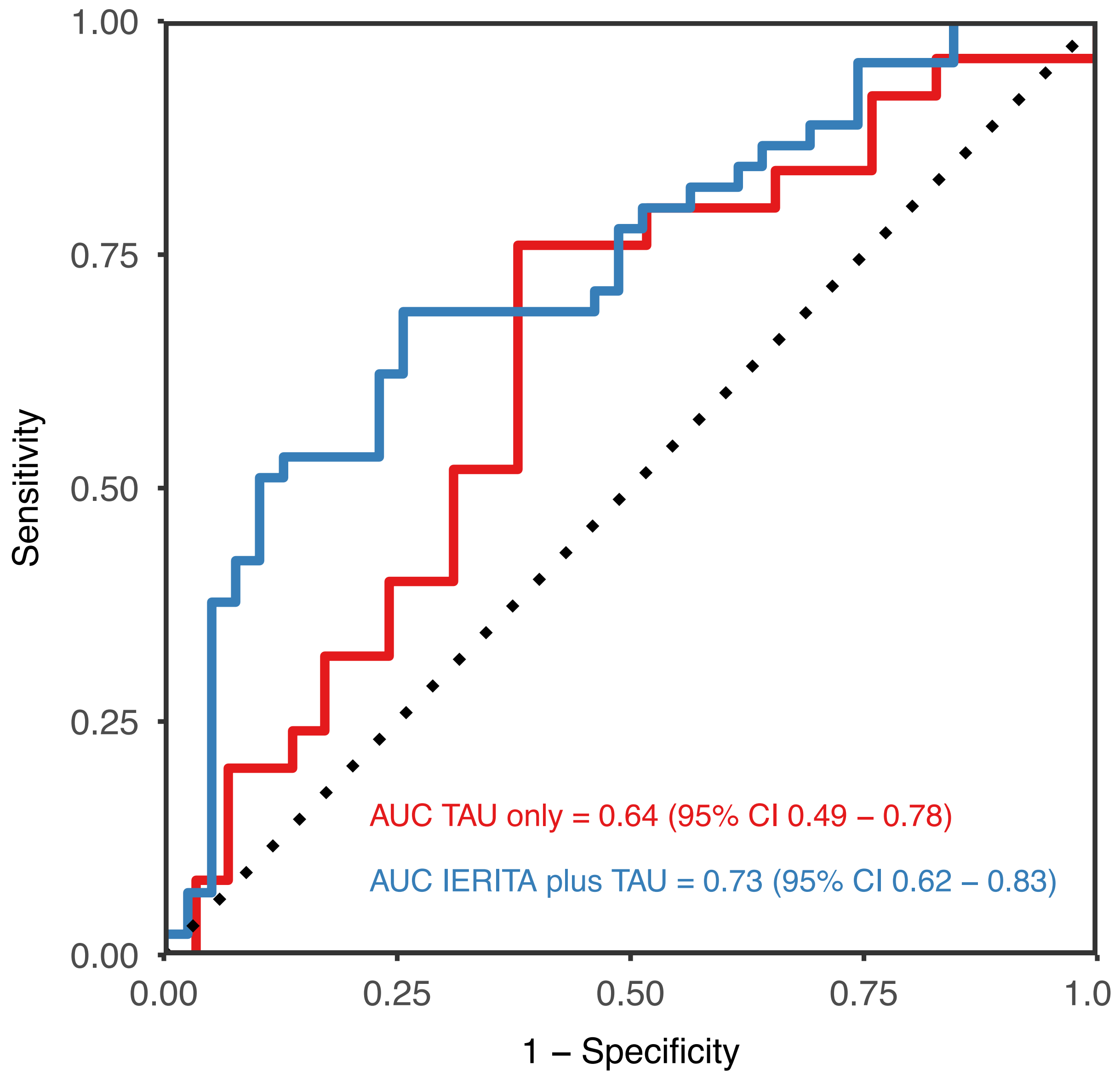

eFigure 1. Receiver operating characteristics curves for ML predictions in the two treatment groups.

References

1. Sheehan DV, Lecrubier Y, Sheehan KH, Amorim P, Janavs J, Weiller E, et al. The Mini-International Neuropsychiatric Interview (M.I.N.I.): the development and validation of a structured diagnostic psychiatric interview for DSM-IV and ICD-10. J Clin Psychiatry. 1998;59 Suppl 20:22-33;quiz 34-57.

2. Philips K. Understanding Body Dysmorphic Disorder: an Essential Guid. Vol. 2009. New York: Oxford University Press;

3. Gratz KL, Dixon-Gordon KL, Chapman AL, Tull MT. Diagnosis and Characterization of *DSM-5* Nonsuicidal Self-Injury Disorder Using the Clinician-Administered Nonsuicidal Self-Injury Disorder Index. Assessment. 2015 Oct;22(5):527–39.

4. Gratz KL, Latzman RD, Young J, Heiden LJ, Damon J, Hight T, et al. Deliberate self-harm among underserved adolescents: The moderating roles of gender, race, and school-level and association with borderline personality features. Personality Disorders: Theory, Research, and Treatment. 2012 Jan;3(1):39–54.

5. Gratz KL. Measurement of Deliberate Self-Harm: Preliminary Data on the Deliberate Self-Harm Inventory. Journal of Psychopathology and Behavioral Assessment. 2001;23(4):253–63.

6. First MB, Gibbon M, Spitzer RL, Williams JBW, Benjamin LS. Structured clinical interview for DSM-IV axis II personality disorders, (SCID-II). Washington, DC: American Psychiatric Association; 1997. In.

7. Guy W, National Institute of Mental, H., Psychopharmacology Research, B., & Division of Extramural Research, P. ECDEU assessment manual for psychopharmacology : 1976. Rockville: National Institute of Mental Health; 1976.

8. Gratz KL, Roemer L. Multidimensional Assessment of Emotion Regulation and Dysregulation: Development, Factor Structure, and Initial Validation of the Difficulties in Emotion Regulation Scale. Journal of Psychopathology and Behavioral Assessment. 2004 Mar;26(1):41–54.

9. Monell E, Birgegård A, Nordgren L, Hesser H, Bjureberg J. Factor structure and clinical correlates of the original and 16-item version of the Difficulties In Emotion Regulation Scale in adolescent girls with eating disorders. J Clin Psychol. 2022 Jun;78(6):1201–19.

10. Neumann A, Van Lier PAC, Gratz KL, Koot HM. Multidimensional Assessment of Emotion Regulation Difficulties in Adolescents Using the Difficulties in Emotion Regulation Scale. Assessment. 2010 Mar;17(1):138–49.

11. Bjureberg J, Ljótsson B, Tull MT, Hedman E, Sahlin H, Lundh LG, et al. Development and Validation of a Brief Version of the Difficulties in Emotion Regulation Scale: The DERS-16. J Psychopathol Behav Assess. 2016 Jun;38(2):284–96.

12. Bohus M, Limberger MF, Frank U, Sender I, Gratwohl T, Stieglitz RD. [Development of the Borderline Symptom List]. Psychother Psychosom Med Psychol. 2001 May;51(5):201–11.

13. Lovibond PF, Lovibond SH. The structure of negative emotional states: comparison of the Depression Anxiety Stress Scales (DASS) with the Beck Depression and Anxiety Inventories. Behav Res Ther. 1995 Mar;33(3):335–43.

14. Osman A, Wong JL, Bagge CL, Freedenthal S, Gutierrez PM, Lozano G. The Depression Anxiety Stress Scales—21 (DASS-21): Further Examination of Dimensions, Scale Reliability, and Correlates. J Clin Psychol. 2012 Dec;68(12):1322–38.

15. Bastien CH, Vallières A, Morin CM. Validation of the Insomnia Severity Index as an outcome measure for insomnia research. Sleep Med. 2001 Jul;2(4):297–307.

16. Chung KF, Kan KKK, Yeung WF. Assessing insomnia in adolescents: Comparison of Insomnia Severity Index, Athens Insomnia Scale and Sleep Quality Index. Sleep Medicine. 2011 May;12(5):463–70.

17. Bond FW, Hayes SC, Baer RA, Carpenter KM, Guenole N, Orcutt HK, et al. Preliminary psychometric properties of the Acceptance and Action Questionnaire-II: a revised measure of psychological inflexibility and experiential avoidance. Behav Ther. 2011 Dec;42(4):676–88.

18. Ravens-Sieberer U, Ravens-Sieberer U, Wille N, Badia X, Bonsel G, Burström K, et al. Feasibility, reliability, and validity of the EQ-5D-Y: results from a multinational study. Qual Life Res. 2010 Apr 17;19(6):887–97.

19. Erhart M, Ottova V, Gaspar T, Jericek H, Schnohr C, Alikasifoglu M, et al. Measuring mental health and well-being of school-children in 15 European countries using the KIDSCREEN-10 Index. Int J Public Health. 2009 Sep;54 Suppl 2:160–6.

20. Fabes, R. A., Eisenberg, N., & Bernzweig, J. Coping with Children's Negative Emotions Scale (CCNES): Description and scoring. Tempe, AZ: Arizona State University; 1990.

21. Shaffer D, Gould MS, Brasic J, Ambrosini P, Fisher P, Bird H, et al. A Children's Global Assessment Scale (CGAS). Archives of General Psychiatry. 1983 Nov 1;40(11):1228–31.

22. Lundh A, Kowalski J, Sundberg CJ, Gumpert C, Landén M. Children's Global Assessment Scale (CGAS) in a naturalistic clinical setting: Inter-rater reliability and comparison with expert ratings. Psychiatry Research. 2010 May;177(1–2):206–10.
